# Distinctiveness and encoding effects in online sentence comprehension

**DOI:** 10.3389/fpsyg.2014.01237

**Published:** 2014-12-12

**Authors:** Philip Hofmeister, Shravan Vasishth

**Affiliations:** ^1^Department of Language and Linguistics, University of EssexColchester, UK; ^2^Department of Linguistics, University of PotsdamPotsdam, Germany; ^3^School of Mathematics and Statistics, University of SheffieldSheffield, UK

**Keywords:** encoding, retrieval, similarity, distinctiveness, sentence processing

## Abstract

In explicit memory recall and recognition tasks, elaboration and contextual isolation both facilitate memory performance. Here, we investigate these effects in the context of sentence processing: targets for retrieval during online sentence processing of English object relative clause constructions differ in the amount of elaboration associated with the target noun phrase, or the homogeneity of superficial features (text color). Experiment 1 shows that greater elaboration for targets during the encoding phase reduces reading times at retrieval sites, but elaboration of non-targets has considerably weaker effects. Experiment 2 illustrates that processing isolated superficial features of target noun phrases—here, a green word in a sentence with words colored white—does not lead to enhanced memory performance, despite triggering longer encoding times. These results are interpreted in the light of the memory models of Nairne, 1990, 2001, 2006, which state that encoding remnants contribute to the set of retrieval cues that provide the basis for similarity-based interference effects.

## 1. Introduction

In everyday life and in laboratory experiments, people remember the unusual better than the usual. Von Restorff's classic findings illustrate this in terms of superior memory for isolated items, such as a bright green word in the context of a list of words colored black (von Restorff, 1933). More generally, a background of homogeneous stimuli favors the recall and recognition of contextually isolated stimuli. These so-called isolation effects share certain key characteristics with another set of memory effects tied to meaning-related processing. The latter include findings that people recall random trivia facts better if they subsequently hear causally-related information (Bradshaw and Anderson, 1982). Word recall and recognition benefits, too, from meaning-related processing (e.g., assessing the pleasantness of word meanings) compared with the processing of superficial features (e.g., identifying whether the word contains the letter “e”), at least under conditions where the memory retrieval phase taps word meaning (Craik and Lockhart, 1972; Hyde and Jenkins, 1973; Craik and Tulving, 1975; Stein et al., 1978).

Although clearly different in some respects (meaning-related processing is not typically taken to be ‘unusual’ or ‘bizarre’), these two sets of effects can be thought of as being parallel in light of their relationship to both encoding and retrieval. In particular, elaboration and isolation each tend to give rise to longer encoding or study times. Elaboration, like isolation, also raises the probability of contextually unique features that serve to differentiate study items at retrieval, because elaboration typically yields highly diagnostic, meaning-related units of information. Thus, these two memory phenomena both potentially reflect a common set of core principles on the encoding-retrieval relationship and the dynamics of retrieval interference.

Correspondingly, mechanistic explanations for both kinds of effects have hinged on processes operative at either the encoding or the retrieval stage. From one view, the mnemonic benefits may arise from increased processing or attention during the encoding phase (Hirshman et al., 1989; Watkins et al., 2000; Shiffrin, 2003), leading to higher fidelity representations, more highly activated representations, or simply a richer set of self-generated features that form a partly redundant network with the core memory representation. This implies a type of investment-reward strategy; by paying for the cognitive costs of “enhanced” representational encoding, the costs of memory retrieval are lessened.

From a different but not mutually exclusive perspective, semantic processing increases the *distinctiveness* of the stimuli at the time of retrieval: “additional conceptual or semantic features help to differentiate the studied words from each other, making these memories less susceptible to interference and/or providing more features that can be cued on a typical recall or recognition memory test” (Gallo et al., 2008, p. 1096; see also Moscovitch and Craik, 1976; Fisher and Craik, 1977; Jacoby and Craik, 1979; Hunt and Worthen, 2006). In other words, semantic processing of words trumps superficial processing because processing a word's meaning generates more contextually unique features than focusing on its sound or orthographic features. For instance, many words in memory may have the sound [aʊ] or the letter sequence “ch.” But relatively few items in memory may be associated with features like “sandy” and “next to the ocean.” Consequently, such accounts predict more than a simple contrast between meaning-related and non-meaning related processing. If semantic processing increases the chances of conceptual distinctiveness, then as semantic processing increases, the chances for successful retrieval from memory should improve up to some arbitrary limit. One implication for at least some such distinctiveness accounts is that a memory target will contrast more with other stimuli, and hence be remembered better, if those *competing* representations elicit more semantic processing. That is, differentiation of two study items may in principle be modulated by the presence/absence of unique semantic features of *either* item, as adding contextually unique features to a competitor cuts down on potential overlap between a competitor and memory target.

Much of this prior research deals with explicit memory for language stimuli, particularly word lists. How linguistic representations are recovered in their most natural setting—online sentence processing—as a function of either elaboration or isolation has not played a significant part in this line of research. This is no doubt due to the implicit nature of memory retrieval during comprehension. Yet comprehending sentences perpetually requires reaccessing some previously perceived information, such as when a pronoun must be interpreted or when the subject of a verb needs to be remembered, and this prior content may vary considerably in the requisite amount of syntactic and semantic processing. Another context in which retrieval from memory happens is in so-called long-distance dependencies (a.k.a. filler-gap dependencies), as in 1:

(1) I finally gave up reading the novel that James Joyce wrote ___ in the 1930s.

To understand this sentence, “the novel” must be retrieved at the embedded verb “wrote” to be properly interpreted as the thematic patient. Evidence that memory retrieval of the argument takes place at the verb comes from reading time data, cross-modal priming tasks, neurophysiological studies, and speed-accuracy tradeoff data (Tanenhaus et al., 1985; Nicol and Swinney, 1989; Kluender and Kutas, 1993; Osterhout and Swinney, 1993; McElree, 2000).

The purpose of the present investigation is to identify whether elaboration and isolation effects occur in online sentence processing and the extent to which such effects might be explained by relating encoding times to retrieval times. The working hypothesis, therefore, is that factors that predict the *success of explict recall* also contribute to the *efficiency of implicit retrieval*. While extant sentence processing models generally ignore variation in the encoding stage as a potential source of processing variation at retrieval sites, cue-based models of retrieval do predict that unique features in a memory target can facilitate retrieval (McElree, 2000; Van Dyke and Lewis, 2003; Lewis and Vasishth, 2005; Lewis et al., 2006; Van Dyke and McElree, 2006, 2011). However, such theories do not make across-the-board predictions that targets with more semantic features, or contextually unique features, ought to be easier to retrieve. This is due to the fact that only those features cued by the retrieval trigger bear on assessments of similarity. For instance, in 2 below, “was complaining” initiates a retrieval probe targeting the animate subject NP “the resident”:

(2) a. The worker was surprised that the resident who was living near the dangerous warehouse *was complaining* about the investigation. [= low interference]
b. The worker was surprised that the resident who said that the neighbor was dangerous *was complaining* about the investigation. [= high interference]

In the high interference condition, the head and dependent are separated by an NP (“the neighbor”) which is a type of semantic object that “can complain” and is also subject marked, similar to the retrieval target. The intervening NP in the low interference condition, in contrast, is inanimate and the object of a preposition, thus mismatching the target semantically and syntactically. Using such materials, Van Dyke (2007) observed evidence of a processing disruption in the high interference condition beginning at the key verbal cluster, which she interpreted in terms of the mechanics of cue-based retrieval. On such an account, features not in the retrieval probe triggered by the verb should have little bearing on memory interference. Whether a target is the only word to begin with an “r” or appears in an unusual font should be immaterial to retrieval efficacy, for example, if verbs do not trigger retrieval probes containing such cues.

In the present experiments, key targets for implicit retrieval in long-distance dependencies differ in the amount of elaboration or “complexity” associated with them (Experiment 1), or with respect to the homogeneity of their text color with the surrounding text (Experiment 2). In both cases, the key features—prenominal modifiers and text color—are unlikely to be directly cued by the retrieval triggering verbs, i.e., verbs don't normally select arguments on the basis of color or the number of modifiers. If elaboration and isolation effects pattern in implicit memory retrieval tasks as they do in explicit memory tasks, then we should expect to see retrieval-related benefits in sentence processing given elaboration or isolation.

Recent reading time data provide some initial evidence that memory retrieval in sentence processing is sensitive to a memory target's representational complexity (Hofmeister, 2011). The term “complexity” is shorthand for the idea that discourse references can differ in semantic complexity via category hierarchy differences, e.g., “a thing” vs. “a stethoscope,” as well as syntactic complexity. For instance, “the landmark on the bluff” encodes both syntactic and semantic features absent in “the landmark.” In clefted constructions like those in 3, participants spent longer reading the head noun of the clefted element as the number of modifiers increased. At the words immediately following the subcategorizing verb (underlined below), however, reading times were faster given more features associated with the target. It is at this subcategorizing verb and the immediately following regions that we expect to observe signs of reactivation and retrieval of the representation in the cleft. Notably, the faster reading times for elaborated conditions do not appear until the subcategorizing verb or shortly thereafter:

(3) a. It was a communist that the members of the club banned from ever entering the premises.
b. It was an alleged communist that the members of the club banned from ever entering the premises.
c. It was an alleged Venezuelan communist that the members of the club banned from ever entering the premises.

Further experiments showed this same pattern even when holding the number of words and syntactic complexity constant, e.g., “which person” vs. “which soldier.” At least in some contexts, therefore, syntactic and semantic processing of linguistic representations facilitates their retrieval from memory. It further suggests that recoverability increases gradiently with semantic processing—something that the list memory literature has so far not shown.

The present self-paced reading studies expand upon these findings in several ways. In Experiment 1, not only the target noun phrase, but also a preceding non-target noun phrase varies in syntactic and semantic complexity. In 4, for example, the matrix object noun phrase is the target for retrieval at “encouraged” and appears in either elaborated or non-elaborated form:

(4) The (senior foreign) diplomat contacted the (ruthless military) dictator who the activist from the United Kingdom encouraged to preserve natural habitats and resources

In addition, the preceding matrix subject noun phrase also varies between an elaborated and non-elaborated form. This manipulation of a competitor's complexity serves two purposes (note: “the activist” serves as a second potential competitor). First, it addresses the previously discussed question of whether elaborative processing linked to non-targets/competitors may facilitate differentiation at retrieval points. Such an idea is plausible from the perspective that providing more detail about any discourse referent or event lowers the chances that it will be confused with some other candidate for memory retrieval. Second, in 4 above, the key retrieval region (“banned”) appears later in the complex sentences than in the simpler ones, opening the door to an explanation based on word position effects. Due to the manipulation of the complexity of multiple phrases in Experiment 1, it will be possible to directly assess whether the effects observable at retrieval sites are reducible to word position effects.

In Experiment 2, the essential components of von Restorff's design are carried over to the domain of sentence processing. Key words in the test sentences are systematically manipulated to make them superficially homogeneous or isolated with the expectation that this will give rise to longer encoding times. The question is whether superficial isolation or differentiation of words in sentences produces retrieval effects that are qualitatively similar to the effects of elaboration in online sentence processing. If they do, then we have evidence of a tight correspondence between implicit and explicit retrieval processes targeting linguistic stimuli.

As we shall see, both elaboration and isolation give rise to longer encoding times, but only the former yields strong evidence for faster reading times at sentence-internal retrieval sites. Moreover, while the elaboration associated with a non-target has striking downstream effects on encoding processes for other discourse referents, the evidence for an effect of non-target complexity on the retrieval of target representations is considerably weaker.

## 2. Experiment 1: target and non-target complexity

### 2.1. Participants

Fifty-two University of Essex undergraduates participated in this study for course credit or payment. All participants identified themselves as native English speakers without significant exposure to a second language before the age of five. No participant data was removed on the basis of accuracy, as all participants scored above 67% correct.

### 2.2. Methodology and materials

In this 2 × 2 self-paced, moving window experiment, 28 items varied in terms of the complexity of a target noun phrase and a non-target noun phrase in the same sentence. Specifically, all sentences contained a transitive matrix clause of the form [NP V NP], where the object noun phrase was modified by an object relative clause. The matrix subject (NP1) appeared with either 0 or 2 modifying words, as did the matrix object NP (NP2), as illustrated below:

(5) a. The congressman interrogated the general who a lawyer for the White House advised to not comment on the prisoners. (= simple simple)
b. The conservative U.S. congressman interrogated the general who a lawyer for the White House advised to not comment on the prisoners. (= complex simple)
c. The congressman interrogated the victorious four-star general who a lawyer for the White House advised to not comment on the prisoners. (= simple complex)
d. The conservative U.S. congressman interrogated the victorious four-star general who a lawyer for the White House advised to not comment on the prisoners. (= complex complex)

The subject of the object relative clause (NP3) was always of the form [DET NOUN]. At the critical embedded verb (“advised” in the example above), proper interpretation of the sentence requires retrieval of the representation referred to by NP2. It is also at such sentence internal retrieval sites that prior psycholinguistic evidence has repeatedly identified signs of similarity-based memory retrieval interference from competing representations (Gordon et al., 2001, 2002, 2006; Van Dyke and McElree, 2006).

Each participant saw only one condition of each item. All sentences were followed by a yes/no comprehension question, and participants received feedback if they answered incorrectly. The comprehension questions targeted information about one of the three referents introduced in the sentence, e.g., “Was the general advised not to comment on the prisoners?” with numerous questions asking about the relationship between two referents, e.g., “Did a photographer embarrass a celebrity?” In Experiment 1, mean comprehension accuracy across all trials, including fillers, was 84% (min = 70%, max = 97%). 70 fillers accompanied the main experimental items for this experiment. Twenty eight of these were from an unrelated experiment.

Materials were presented and randomized with the reading time software linger v. 2.94, developed by Doug Rohde (available at http://tedlab.mit.edu/~dr/Linger/). The experimental items were randomized by the experimental software, and at least one filler separated each critical item. At the beginning of each trial, a fixation cross at the left of the screen appeared on the same line where the target sentence subsequently appeared. On pressing a key, the cross disappeared and the first word of the sentence was shown. Words not currently being read were not presented on screen and were not masked with dashes, i.e., the screen was blank except for the word currently being read. We opted for this method to prevent participants from using end-of-sentence information to modulate their reading rate, since the target sentences differed in overall length.

Prior to statistical analysis, raw reading times greater than 5000 ms or less than 100 ms were removed, affecting a total of 0.001% of the data. No additional outlier removal processes were performed. All data were analyzed regardless of comprehension accuracy in order to capture any reading time differences that may reflect memory retrieval failures. In other words, as we are investigating not only retrieval efficiency but also success, excluding trials that were incorrectly responded to would eliminate an important and relevant subset of the data on which retrieval of the target NP potentially failed. However, in the Supplementary Materials, we also present secondary analyses using only data from correctly answered trials.

**Figure 1:**
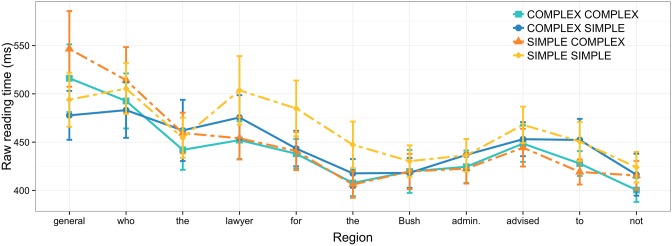
**Raw reading times in Experiment 1 by region; error bars show 95% confidence intervals**.

Reading times were log-transformed to normalize the residuals and reduce the effect of extreme data points. Then, the log reading times for all stimuli (fillers included) were regressed against several predictors known to affect reading times in self-paced reading tasks: word length and log list position (Ferreira and Clifton, 1986; Hofmeister, 2011). Specifically, longer words predict longer reading times and later list positions predict faster reading times as participants progress through the experiment. The model estimating these effects included a random effects term for participants, i.e., by-participant random intercept adjustments. We used data from fillers in this process to produce maximally general estimates of word length and list position. The residuals of this model—residual log reading times—are the dependent variable analyzed here (Figure 1 shows raw reading times to provide a more interpretable scale for the effects). All categorical predictors variables were sum coded to reduce effects of collinearity.

All analyses were conducted with Bayesian hierarchical models, fit with Stan and the R package rstan. We employed these models because they allow us to fit complex hierarchical models with maximal random effect structures that often do not converge using other popular linear regression packages such as lme4. Moreover, as noted in Husain et al. (2014), using Bayesian models allows us to assess and compare the weights of evidence for particular hypotheses. This means that we avoid categorizing effects as significant or non-significant, eschewing traditional statistical inference based on *p*-values. Instead, we make statistical inferences for particular hypotheses by computing the posterior probabilities for relevant parameters θ_*i*_ by sampling from their posterior distribution.

Each word region model used 4 chains, 5000 samples per chain, a warm-up of 2500 samples, and no thinning, resulting in 10,000 samples for each parameter estimate. All models contained fixed effect parameters for NP1 complexity, NP2 complexity, and their interaction. They also included by-participant random intercept adjustments and random slopes for all fixed effect terms (3 parameters), and by-item random intercept adjustments and random slopes for NP1 complexity, NP2 complexity, and their interaction (3 parameters). We utilized weak, uninformative priors for all key parameters, including participant and item adjustments. For each model, P(θ|*data*) indicates the probability that the parameter estimate is negative, i.e., speeding up occurs. For instance, an estimate that P(θ_*complex*_ < 0) = 0.99 signifies that we can be 99% certain that complexity speeds up reading; in contrast, if P(θ_*complex*_ < 0) = 0.01, we can infer with 99% certainty that complexity slows down reading. These probabilities were obtained by calculating the percentage of posterior samples above or below zero. To improve readability we will write P(θ < 0) for P(θ < 0|*data*).

Three regions are analyzed in Experiment 1: the head noun of NP2, the head noun of NP3, and the verb that subcategorizes for NP2. As reading time effects in self-paced reading experiments often spill over onto subsequent words, results for the word regions immediately after the relevant sites are also reported. No significant effects of the experimental manipulations on comprehension accuracy were found so they are not discussed here (see data in Supplementary Materials).

### 2.3. Results

#### 2.3.1. NP2 head noun

As shown in Table 1, greater syntactic and semantic complexity of NP2 leads to longer reading times at this region. Greater complexity of NP1, however, has a weaker effect in the opposite direction. That is, reading times at the NP2 head noun were somewhat faster when NP1 was complex, compared to when it was syntactically and semantically simple. There is no compelling evidence for an interaction at this word region.

**Table 1:** **Model summary for Experiment 1 for each region and fixed effect factor**.

**Region**	**Factor**	**Mean**	**CrI lower**	**CrI upper**	**P(β < 0)**
Head noun	NP1 complexity	−0.012	−0.033	0.009	0.866
	NP2 complexity	0.038	0.011	0.065	0.002
	NP1 × NP2 complexity	−0.003	−0.024	0.017	0.621
RC subject head noun	NP1 complexity	−0.005	−0.025	0.014	0.702
	NP2 complexity	−0.022	−0.039	−0.005	0.996
	NP1 × NP2 complexity	0.002	−0.016	0.020	0.397
RC subject head noun + 1	NP1 complexity	−0.014	−0.032	0.002	0.955
	NP2 complexity	−0.012	−0.027	0.002	0.951
	NP1 × NP2 complexity	0.013	−0.002	0.027	0.043
RC verb	NP1 complexity	−0.005	−0.019	0.010	0.757
	NP2 complexity	−0.014	−0.027	−0.001	0.979
	NP1 × NP2 complexity	0.011	−0.005	0.025	0.079
RC verb + 1	NP1 complexity	−0.004	−0.009	0.017	0.281
	NP2 complexity −0.019	−0.019	−0.035	−0.004	0.991
	NP1 × NP2 complexity	0.006	−0.007	0.019	0.200

*Summary includes the posterior 95% Credible Interval (CrI), i.e., the lower CrI refers to the 2.5% bound and the upper CrI refers to the 97.5% bound. P(β < 0) indicates the probability that complexity slows reading times effects, i.e., values closer to 0 indicate slowing down and values closer to 1 indicate speeding up due to complexity*.

#### 2.3.2. NP3 head noun + spillover

Complexity of NP2 also has an effect on reading times at the head noun of NP3 (e.g., “lawyer”): reading times are faster when NP2 is relatively complex. At the word immediately following the head noun (“for” in 2.2), an interaction of NP1 & NP2 complexity arises, along with main effects of NP1 & NP2 complexity. This interaction stems from the fact that NP1 complexity leads to faster reading times only when NP2 is simple.

#### 2.3.3. Relative clause verb + spillover

A main effect of NP2 complexity is evident at the critical relative clause verb: when NP2 is complex, reading times are faster than when NP2 is simple. Alongside this main effect, the results provide weak support of an interaction due to the fact that the complexity of NP1 affects reading times more when NP2 is simple. Put differently, there is no added processing facilitation due to the complexity of NP1 when NP2 is itself complex. The NP2 complexity effect also carries over onto the word immediately after the verb. In fact, the effect is even more pronounced at this region. Here, signs of an interaction are considerably weaker, as illustrated in Figure 1.

#### 2.3.4. Correctly answered trials only

**Figure 2:**
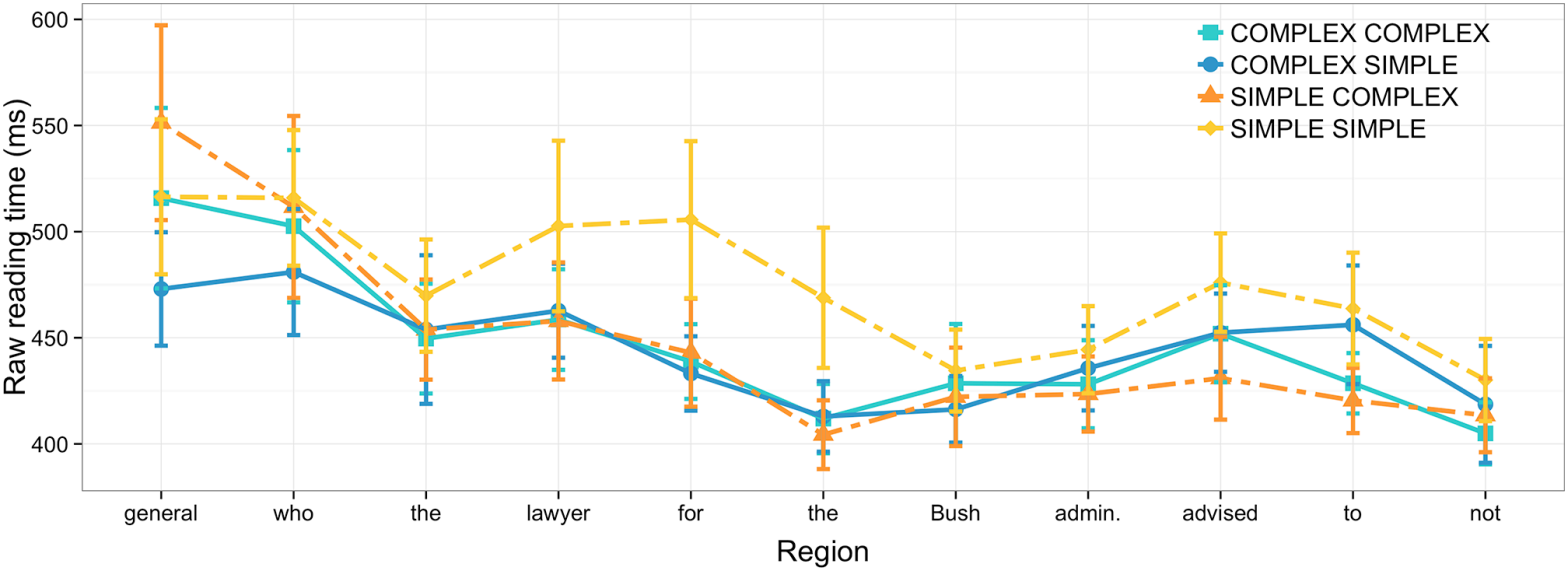
**Raw reading times in Experiment 1 for correctly answered trials; error bars show 95% confidence intervals**.

We conducted secondary, *post-hoc* analyses using only data from correctly answered trials to determine whether the observed complexity effects were tied to trials where participants answered incorrectly. As depicted in Figure 2, all main findings persist in this data subset with NP2 complexity effects at the NP3 and the relative clause verb slightly increasing in magnitude.

### 2.4. Discussion

When readers encode additional syntactic and semantic features, they read faster at sentence-internal retrieval sites. This pattern holds, however, primarily for NP2—the downstream retrieval target. At the relative clause subject, reading times are faster when NP2 is syntactically and semantically complex, and this effect re-emerges at the retrieval triggering verb, continuing on into the spillover region.

Effects tied to NP1—the preceding non-target—are comparatively weaker and tied to the status of NP2. Whereas the effects of the complexity of NP2 show up at the head noun of NP3, the impact of NP1 complexity does not emerge until the head noun's spillover region. More tellingly, NP1 complexity affects reading rates selectively: only when NP2 is simple, and hence syntactically similar to NP3, does greater NP1 complexity reduce reading times. At the retrieval region, too, effects of the complexity of NP1 are weak compared to those of NP2. While there are hints at the relative clause verb that NP1 complexity has some facilitatory effects, such effects (1) do not have the duration of those tied to NP2, (2) are statistically weaker, and (3) only appear when NP2 is simple. In essence, differences in the feature-based complexity of a competitor do not weigh as significantly on retrieval in sentence comprehension as differences in target complexity. This suggests rather specific constraints on the dynamics of encoding and retrieval with respect to the computation of similarity-based interference in sentence processing that are dealt with in the General Discussion.

Two notable conclusions can be drawn from these results. First, word position alone cannot account for the reading time differences at the retrieval sites. Inside the relative clause, the complex-simple and simple-complex conditions match each other with respect to word position, yet display different profiles at the word following the subcategorizing verb. Moreover, if elaboration effects at the retrieval region owe their existence to a basic linkage between word position and reading rate, then we would expect the reading times for the conditions to be ordered according to word position. However, the complex-complex condition proved to be no faster than the simple-complex, despite the retrieval region appearing two words later in the sentence. Second, the lack of a main effect of NP1 complexity at the retrieval region argues against a general preference for maximal descriptiveness. Indeed, nowhere in the sentence does there seem to be a notable advantage for modifying both NPs in the matrix clause. As noted above, however, NP1 complexity does impact the processing of NP3 when NP2 is simple. We take this to mean that encoding interference arises at NP3 when all the NPs match in form, but altering the form of either of the preceding NPs mitigates these interference effects.

A valid concern with respect to these data concerns the relationship between the effects at NP3 and the verb. Are these separate effects, or do the effects at the verb simply reflect extended spillover effects that originate with processing NP3 in the above stimuli? This concern is amplified by signs of NP2 complexity effects at the region before the retrieval-triggering verb. Several arguments, however, speak against the interpretation that the differences at the verb and its spillover region reflect a continuation of previously initiated processes. First, a separate analysis revealed that the NP2 complexity effect at the verb remains intact even after including reading times from the word before the verb as a covariate ($\hat{\mu }$ = −0.022; CrI Lower = −0.038; CrI Upper = −0.006: P(β < 0) = 0.997). Second, consideration of only correctly answered trials shows that the effects at the verb are magnified, while differences at the preceding region are minimized (see Supplementary Materials for model summaries). Some of the variation across conditions immediately prior to the verb thus comes from trials where encoding or retrieval processes may have been compromised. Further supporting this interpretation, it was found that several poorly-performing participants (who averaged 56% correct on the critical trials) were the primary source of reading times differences at the word region preceding the verb. In the case of these participants, it is indeed possible that encoding difficulties continued on into the retrieval region^1^. Taken together, these observations support the interpretation that the effects at the verb and subsequent word reflect cognitive processes that begin at the verb.

## 3. Experiment 2

If complexity effects arise during sentence processing because additional semantic or conceptual features distinguish representations from one another, this raises the question of whether all types of unique features distinguish comprehension-based representations. There may be nothing special, mnemonically speaking, about syntactic and semantic features in comprehension. Experiment 2 consequently looks at whether unique features in general stimulate faster processing at retrieval sites in comprehension. But this experiment also has a secondary purpose. In Experiment 1, longer encoding times match up with shorter reading times at or directly after the retrieval site. Thus, one take on the previous results is that additional semantic features stimulate more processing, which facilitates downstream retrieval. By manipulating the homogeneity of superficial features in Experiment 2, we address both issues due to the expectation that isolated word stimuli will not only generate contextually unique features (by definition), but will also lead to extended processing times during the encoding phase. The question is how this will bear, if at all, on the processing of words that trigger the retrieval of these encodings.

### 3.1. Participants

Forty-four UC-San Diego students participated in this study, in exchange for course credit. All subjects identified themselves as monolingual American English speakers without any known history of color blindness. The results from two participants were removed due to comprehension question accuracies below 67%.

### 3.2. Methodology and materials

Thirty-two items were constructed with an object noun phrase in a transitive main clause modified by an object relative clause, as in 6 below. Textually, the conditions were identical to each other.

(6) The congressman interrogated the **general** who the lawyer for the Bush administration advised ___ to not comment on the detainees.

To manipulate processing during the encoding phase, the head noun of the object NP (“general” above) appeared either in the same color as the surrounding sentence text (white), or else in an incongruent color (bright green). Additionally, the color of the word that triggered retrieval (“advised”) also varied between congruent and incongruent. This second manipulation provides a needed check to ensure that participants do not read later word regions faster because of anticipation for an incongruently colored word. Moreover, in the condition with the green head noun and green verb, we can assess whether reinstating features of the encoding phase aids in retrieval. Hence, each item had four conditions (white-white, white-green, green-white, green-green), but each subject saw only one condition of each item.

Participants received instructions that the color of the words in the sentences was immaterial to the task and that they did not need to respond to color changes. Yes/no comprehension questions followed each item, and participants received negative feedback if they answered a question incorrectly. Sixty fillers accompanied these critical items: 20 with 0 green words, 20 with 1 green word, and 20 with 2 green words. For filler items with 1 green word, the word was randomly selected from all words in the sentence. For fillers with 2 green words, one appeared randomly in the the first half of the sentence and the other in the second half. All fillers had a syntactic structure different from that used in the critical items.

The materials were presented in a self-paced, center presentation paradigm via a propriety software package. Only one version of each item appeared on each of four experimental lists, whose contents were pseudo-randomized such that at least one filler intervened between each critical item. A fixation cross in the center of the screen appeared before each trial, and a comprehension question followed every experimental trial, including fillers. Participants received feedback only on incorrectly answered trials.

The outlier removal process, computation of residual log reading times, and Bayesian analysis procedure all followed those used in Experiment 1. As in that experiment, there were no differences in comprehension accuracy (green-green = 76%, green-white = 77%, white-white = 76%, white-green = 76%). Here, we analyze residual log reading times at the head noun of the matrix object phrase and the relative clause verb that triggers its retrieval.

### 3.3. Results

**Figure 3:**
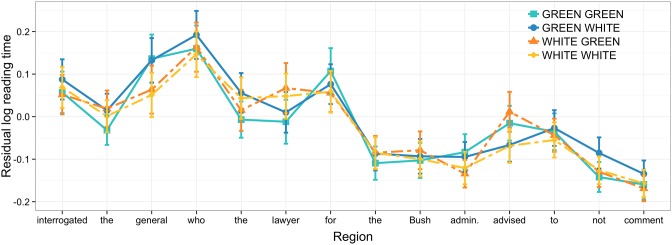
**Residual log reading times at verb in Experiment 2; error bars show 95% confidence intervals**.

At the object head noun, incongruent, green words slow reading times, compared to the congruent, white words (see Figure 3). Similarly, looking at reading times at the retrieval region [“advised” in (3.2)], a perceptually incongruent, green verb slows reading speed compared to a congruent, white one.

In contrast to the pattern observed in Experiment 1, the increased encoding time at the object head noun due to superficial incongruence leads to relatively weak facilitation effects at the retrieval site, as shown in Table 2. In fact, the mean parameter value resides less than one standard deviation (=0.011) from zero, according to the model results. The mean value for the condition where both the noun and the verb are incongruently colored reflects slightly faster reading than for the condition where only the verb is incongruent (green-green: −0.015, *SE* = 0.021; white-green: 0.011, *SE* = 0.024). This difference of roughly one standard error is why the model acknowledges a relatively weak effect of noun color (and an interaction with verb color) on reading times at the verb. At regions after the verb, there is no evidence that processing an incongruently colored target noun facilitates processing.

**Table 2:** **Model summary for Experiment 2**.

**Region**	**Factor**	**Mean**	**CrI lower**	**CrI upper**	**P(β < 0)**
Head noun	Noun color	0.039	0.007	0.071	0.008
	Verb color	0.005	−0.025	0.034	0.362
	Noun color × Verb color	−0.001	−0.031	0.028	0.532
RC verb	Noun color	−0.007	−0.029	0.016	0.731
	Verb color	0.032	0.008	0.056	0.001
	Noun color × Verb color	−0.008	−0.034	0.017	0.734

*Summary includes the posterior 95% Credible Interval (CrI), i.e., the lower CrI refers to the 2.5% bound and the upper CrI refers to the 97.5% bound. P(β < 0) indicates the probability that incongruence slows reading times effects, i.e., values closer to 0 indicate slowing down and values closer to 1 indicate speeding up due to incongruence*.

### 3.4. Discussion

Increased processing times triggered by incongruent stimuli at the encoding site had weak effects on processing at the retrieval site when compared to the complexity effects observed in Experiment 1. Only when the relevant perceptual features were reinstated at the retrieval site was there any numerical retrieval advantage for perceptually incongruous stimuli. Even in this case, the facilitating effects were quite mild and would be deemed insignificant on classical frequentist methods of analysis. These findings imply that contextually unique features do not necessarily lead to improved memory performance, nor does increased processing time.

These findings may initially seem to contrast with memory results for recognition/recall of items presented in lists. For instance, von Restorff (1933) observed better recognition for words that appeared in superficially incongruent states. Similar findings of improved memory performance for superficially incongruent linguistic items (within mixed lists, but not unmixed lists) appear in Bruce et al., 1976, Hunt and Elliot, 1980, Hunt, 1995, Dunlosky et al., 2000, *inter alia*.

However, the current evidence reinforces the idea that the memory retrieval context is of utmost importance—a point frequently reiterated by memory researchers such as Tulving, Nairne, and others. In the present case, color or other superficial orthographical features rarely matter in written, sentence comprehension. Particularly if subjects are requested to ignore such information, there is little reason for subjects to recruit such potentially distinctive features in memory retrieval, whether or not they elicit more processing. In contrast, standard list recall or recognition tasks are novel encoding and retrieval contexts for participants—we are not standardly shown a list of words and then asked to retrieve them later, so we have few if any entrained habits. Consequently, in such novel circumstances, participants reasonably utilize all manner of perceptual features in recovering representations from memory.

In short, this experiment establishes that the uniqueness effects in language comprehension depend heavily on the retrieval context. What counts as unique critically depends on the nature and demands imposed at the retrieval site. Ultimately, if some set of representational features are unimportant for memory retrieval, then their congruence with other local feature appears to also have little import for memory retrieval.

## 4. General discussion

Increased processing during the encoding phase leads to more efficient retrieval processing in sentence comprehension, but only under certain conditions. Experiment 1 illustrated that increased processing associated with the downstream target benefits retrieval-related processing, whereas processing related to non-targets had relatively weak, short-lived effects that only arose when the target itself was not elaborated. Experiment 2 expanded on this by showing that not just any sort of extra processing facilitates memory (even for targets)—indeed, the results suggest that it is not about processing *per se* so much as the role of the features themselves in the retrieval process. In many respects, these results parallel the findings of studies assessing the effects of elaboration on long-term memory performance for linguistic stimuli (Stein et al., 1978; Eysenck, 1979; Jacoby and Craik, 1979; Reder, 1980; Bradshaw and Anderson, 1982; Reder et al., 1986; McDaniel et al., 1988). At the same time, they add to these studies by showing that memory performance improves as meaning-related processing increases for linguistic stimuli in the context of sentence comprehension. Secondly, they demonstrate that these effects occur even in covert retrieval settings, where the time constraints of real-time comprehension limit the options for retrieval strategies. Third, the results from the final experiment demonstrate that unique representational target features and increased processing do not always lead to improved memory retrieval.

Both sets of findings—the advantage of additional processing for targets compared to non-targets, and the fact that increased processing time does not necessarily benefit memory retrieval—can be understood through the lens of the short-term, feature-based retrieval model of Nairne (1990, 2001, 2006), with some minor new assumptions (several other memory models make similar predictions, e.g., Oberauer and Kliegl, 2006 and Shiffrin, 2003, although the details differ). In Nairne's model, memory items are represented as a vector of features, e.g., [C X 1 2 3]. Retrieval cues consist of lingering, typically blurry, records of the immediate past, e.g., [C X ? 2 3], as well as cues from the local retrieval context. In turn, these two sets of cues form a memory probe that is compared against a set of candidate memory items. The ultimate objective is to “redintegrate” the retrieval cues with a memory item, as the cues by themselves cannot be directly interpreted (Ericsson and Kintsch, 1995). The probability of retrieving an event E_1_, given a retrieval probe X_1_ depends upon the similarity or feature-overlap of X_1_ and E_1_, as well as the similarity of X_1_ to other memory candidates:

(1) $$P_{r}(E_{1}|X_{1})=\frac{s(X_{1},E_{1})}{\sum s(X_{1},E_{n})}$$

The similarity between a memory item and a retrieval probe is determined by the number of mismatching features divided by the total number of compared features (*d*):

(2) $$s(X_{1},E_{1})=e^{-d(X_{1},E_{1})}$$

Because retrieval probes consist of remnants of the original encoding process that need to be interpreted by comparing them against candidate memory items, any contextually unique features in a target will improve the chances for successful retrieval. In short, a target's recoverability increases if it possesses a feature that no other competitor shares.

Nairne (2006) employs this model to explain isolation or distinctiveness effects, since odd/bizarre items possess features that mismatch with the features of some homogeneous background set. For instance, imagine a context where the original encoding is perfectly intact and acts as the sole source of retrieval cues, e.g., X_1_ = E_1_. Any contextually unique features will increase the dissimilarity or mismatch between the retrieval cues and competitors, even though contextual uniqueness does not directly affect the similarity value between the target and retrieval probe.

An implied consequence of such a theory is that simply adding features to a target is predicted to increase the odds of sampling from memory, so long as these features are unique. Table 3 shows how the probability of sampling a target increases as the number of mismatching features between the target and non-targets increases, even though the number of shared features remains constant (see Hofmeister et al., 2013 for an application of this model to the processing and acceptability of multiple wh-questions in English). The added features Q, R, & N in the undegraded probe lack any correlates in the competitors, meaning that the mismatch between them and the probe increases, effectively upping the chances for sampling the target.

**Table 3:** **Similarity values and predicted sampling probabilities for two retrieval contexts**.

**Cue**	**Traces**	**Similarity**	**Samp. prob**.
[C C 2 3 1]	[C C 1 2 3]	0.55	0.26
	[C C 2 3 1]	1.0	0.48
	[C C 3 1 2]	0.55	0.26
[C C 2 3 1 Q R N]	[C C 1 2 3]	0.47	0.24
	[C C 2 3 1 Q R N]	1.0	0.52
	[C C 3 1 2]	0.47	0.24

**Figure 4:**
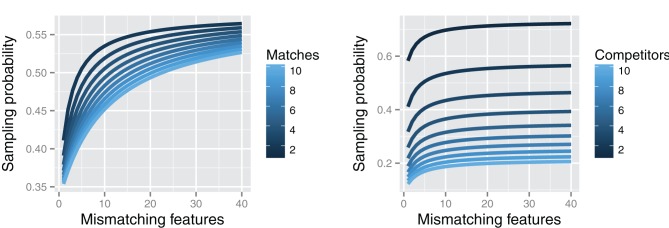
**Left:** Relationship between number of unique target features (mismatching with non-targets) and average sampling probability of target with two competitors. In descending order, the lines show the varying sampling probability curves for 2 to 10 probe features matching with each competitor. **Right:** Relationship between number of unique target features and average sampling probability of target as a function of the number of competitors (from 1 to 10 in descending order), assuming two matching features between the probe and each competitor. The retrieval sampling curves illustrate the diminishing effects of mismatching features and the relatively greater effect of the number of competitors compared to the number of matching features.

As Figure 4 illustrates (left panel), the effect of adding mismatching or contextually unique features faces some restrictions: increasing the number of mismatches yields diminishing returns, ultimately asymptoting at a level that depends upon the number of features involved and the number of feature matches. In less formal terms, adding a little unique, diagnostic information can be quite helpful for memory retrieval, but adding lots of unique information is not likely to contribute much more. This model also predicts that the number of competitors affects retrieval probability much more dramatically than the number of overlapping features. On the right side, Figure 4 shows that going from one competitor to three competitors which each share two features with the probe nearly halves the chances of retrieval. In contrast, the difference between two competitors with 2 vs. 10 matching features never exceeds 10% (see left side of Figure 4).

A key component of this type of model is that a fragile copy of the original encoding process stored in primary memory provides a source of retrieval cues. This makes explicit the idea that syntactic and semantic features not directly invoked by the local sentence context can influence retrieval processes, in contrast to assumptions that only the similarity of features “grammatically derived from the current word and context” enter into considerations of similarity-based interference (Lewis et al., 2006, p. 448)^2^. Sentence processing models built upon the latter kind of assumption face difficulty explaining some classic retrieval interference effects in the sentence processing literature (Logačev and Vasishth, 2012). For instance, Gordon et al. (2001) show that processing in object-cleft sentences like 7 is easier at the subcategorizing verb (“saw”) when the two NPs are of different types (proper name vs. definite description), but that such effects are absent in subject relativization constructions:

(7) It was John/the barber that the lawyer/Bill saw in the parking lot.

These effects are commonly understood in terms of similarity-based interference: if the target noun phrase overlaps in form with another local noun phrase that appears before the verb, memory retrieval difficulty ensues, ostensibly because the retrieval cues match multiple memory representations. As the second NP occurs after the verb in subject relatives, no possibility for interference exists. Notably, the verb triggering retrieval (“saw”) does not itself supply cues as to the nominal type of the clefted element; indeed, no language appears to explicitly code whether a verb requires a lexical, pronominal, or some other type of nominal argument. So, if the similarity effects arise because retrieval cues match multiple representations, then those cues must come from a source besides the verb. The original encoding of the target provides the most obvious source of such cues. Not only does this open up a way to explain similarity-based effects due to overlapping referential form, it can also accommodate phonological similarity effects such as the observed reading time contrast at the embedded verb in sentences like “The baker that the banker sought found the house” vs. “The runner that the banker sought found the house” (Acheson and MacDonald, 2011)^3^.

The current findings add a further data point to our developing picture of similarity-based interference in sentence processing: non-target distinctiveness has a weaker role to play in retrieval interference than target distinctiveness. These effects can be straightforwardly accommodated with some specifications about how similarity is calculated. Following Nairne (2006), let's assume that similarity at retrieval sites is calculated by establishing mismatches with the lingering features of a target's encoding remnant and any other features in the retrieval probe. A memory probe such as [C X 1 2 3] will mismatch equally with a competitor representation like [C X 4 5 6] as [C X 4 5 6 L M], e.g., 3 out of 5 probe features will mismatch with competitor features. In other words, it is the number of features in the probe that determine how many mismatches there can be, and not the number of features in a memory retrieval candidate. Adding unique features to some non-target, therefore, will not directly affect the probability of sampling the target because it does not contribute to the set of retrieval cues.

The data hint nonetheless at some retrieval effects linked to the elaboration of non-targets, specifically when the retrieval target itself was syntactically and semantically simple. This would seem to initially contradict the above view that the uniqueness of non-targets does not directly bear on retrieval efficiency. There is no contradiction, however, if these non-targets effects are byproducts of encoding interference. That is, we presume that the uniqueness of features in non-target nominals affects how other local nominals, including downstream targets, are encoded, and indirectly influence retrieval operations *via such encoding effects*. Even more generally, encoding interference feeds into retrieval interference.

Already, evidence exists that similarity between linguistic representations in memory and those being encoded can lead to processing disruptions, during both encoding and retrieval stages (Gordon et al., 2002; Acheson and MacDonald, 2011). For example, Gordon et al. (2002) provide evidence of reading slowdowns when words on a sentence-external memory list are similar to key words inside the sentence, e.g., proper names vs. definite descriptions, both at the encoding site for the sentence-internal words and later at retrieval sites for those same words. We would add to this by hypothesizing that encoding interference may contribute to the degradation of memory representations, following research that suggests that forgetting in short-term memory for linguistic representations can stem from feature overwriting (Oberauer and Lange, 2008; Oberauer, 2009). Because these features that are susceptible to overwriting also contribute to retrieval cues on the account sketched above, feature loss could compromise any cue-based retrieval process.

Applying these hypotheses to the results of Experiment 1, encoding interference emerges as an indirect (and accordingly, weaker) contributor to retrieval differences, beyond what is predicted by the model of memory retrieval inspired by Nairne. Specifically, similarity between the referring expressions determines encoding interference, which can affect the integrity of the trace for the target nominal. So, when NP1 is complex and NP3 is simple or vice versa, this translates to a reduced danger of feature overwriting, compared to when they are both simple. In turn, the potential for retrieval interference is mitigated when the two initial NPs mismatch in complexity, because the trace for NP2 is more likely to be intact. Things are somewhat more complicated when NP1 and NP2 are both complex: while overlapping in structural form, the NPs carry more unique semantic features than their simpler counterparts. In this case, we tentatively take the results to mean that encoding interference is relatively low, compared to the case where both NPs are simple, but not any lower than when just one such NP is complex. These ideas require further tests to be substantiated, as the current experiments were not designed to test them. Nonetheless, we maintain that the relatively weak effects of non-targets can best be explained by appealing to the effect of encoding interference on memory retrieval.

Notably, redintegration-based models of memory do not require that every perceivable feature matters for memory retrieval. Listeners or readers may preferentially not encode some features in typical language settings, such as modality-specific features or exclude such features from the retrieval probe based on prior experience of the efficacy of such features. The advantage of increased processing thus depends upon the discourse context and the extent to which processing engenders unique features that come into play during the retrieval stage. From this perspective, encoding manipulations cannot have a predictable effect on memory in the absence of information about the encoding and retrieval contexts—what other memory candidates are available and what the retrieval cues are.

The results of Experiment 2 align with this perspective, in light of the absence of isolation or superficial processing effects. Modality-dependent features, such as orthography, font style, text color, etc., often play a large role in various laboratory tests of memory and in effects such as the auditory recency effect, but they appear to have a lesser role in guiding retrieval in sentence processing contexts. Such contrasts, though, are explicable in terms of task demands and prior experience. Word recall and recognition tasks lie outside the typical range of personal pastimes, whereas sentence comprehension is an everyday occurrence. This arguably leads participants to utilize a wider range of possible retrieval cues in word recall tasks, whereas prior experience with sentence processing would bias against the use of modality-specific features to distinguish memory representations. Instead, modality-independent features—properties that largely remain constant across presentations or modalities such as syntactic category and meaning—provide the basis for restoring linguistic representations during sentence processing because of their diagnostic potential. Thus, it is due to the fact that discrimination between language representations in sentence comprehension depends on syntactic and semantic features that the uniqueness of these features bears on determinations of retrieval ease and success. Correspondingly, the primary source of retrieval difficulty in language comprehension—overlapping semantic and syntactic representations and the resulting interference—is what gives additional linguistic processing mnemonic value, and why other types of processing such as superficial processing have little mnemonic value.

## 5. Conclusion

These tests of implicit memory establish that elaboration effects occur in online sentence processing tasks, as they do in explicit tests of memory. In Experiment 1, we found that increased processing of syntactic and semantic features connected to the target benefits memory retrieval in sentence processing; however, additional processing directed toward non-targets had substantially weaker effects on processing at retrieval sites. In Experiment 2, it was established that the processing of superficial features or features connected to non-targets yielded insubstantial processing advantages at retrieval sites, despite leading to longer encoding times. As sentence processing demands differ from those of explicit memory tasks, it is unsurprising that the effects of encoding manipulations can differ drastically across tasks with inherently different retrieval contexts. This apparent dynamic interaction between encoding and retrieval led Tulving (1983, p. 239) to argue against any statements of the form that “encoding operations of class X are more effective than encoding operations of class Y” (see also Neath and Surprenant, 2005 for a recent review). In short, encoding manipulations are unpredictable without additional information about the nature of the retrieval task and the background of competing representations.

The comparison of memory findings in the broader psychology and psycholinguistics literature also led to a unified theoretical account of distinctiveness effects, applicable across tasks. Capturing the interplay between representational uniqueness and retrieval probability, Nairne's feature-based model provides a means for introducing retrieval cues that are unlikely to be cued by local grammatical memory triggers via the use of a fragile copy of the original encoding. This fills a critical gap in cue-based models of retrieval in sentence processing by pointing to alternative sources of retrieval cues beyond the local context, thus accounting for a variety of otherwise unexplained similarity-based effects in sentence processing.

### Conflict of interest statement

The Associate Editor Claudia Felser declares that, despite being affiliated to the same institution as the author Shravan Vasishth, the review process was handled objectively and no conflict of interest exists. The authors declare that the research was conducted in the absence of any commercial or financial relationships that could be construed as a potential conflict of interest.

## Footnotes

^1^This might be taken as justification to exclude these participants altogether; however, we see no reason to exclude participants because they encounter more encoding problems or read less accurately than their peers.

^2^Current sentence processing models are not without means to explain effects of complexity on memory retrieval. For instance, on the ACT-R-based theory of Lewis and Vasishth (2005), processing syntactic material that modifies some previously constructed representation requires the restoration of the stored memory item. This retrieval process, in turn, raises the overall activation level of the item, making it easier to retrieve subsequently. Thus, complexity-based effects on retrieval emerge most straightforwardly as the byproduct of encoding processes. Moreover, additional study time potentially allows for more accurate encoding, providing greater chances that target features will be cued at the retrieval site (see also Shiffrin, 2003). However, as retrieval cues are limited to those provided by local grammatical context, there is no guarantee that unique semantic or syntactic features will factor into estimates of similarity and thus retrieval difficulty.

^3^Acheson and MacDonald (2011) illustrate similar effects in subject relatives, as well, suggesting that phonological similarity gives rise to encoding interference and not simply retrieval interference.
