# Supplementary material for: Distinctiveness and encoding effects in online sentence comprehension
Source: Front Psychol. 2014 Dec 12;5:1237. doi: 10.3389/fpsyg.2014.01237 (PMC4264409; doi:10.3389/fpsyg.2014.01237)
Supplement: Supplementary file 1 [file DataSheet1.PDF]

1 APPENDIX

Table 1. Model summary for correctly answered trials only from Experiment 1 for regions from one word before relative clause verb to one word after. Summary includes the posterior 95% Credible Interval (CrI), i.e., the lower CrI refers to the 2.5% bound and the upper CrI refers to the 97.5% bound. P($\beta <$ 0) indicates the probability that complexity slows reading times effects, i.e., values closer to 0 indicate slowing down and values closer to 1 indicate speeding up due to complexity.

Region	*Factor*	*Mean*	*CrI lower*	*CrI upper*	*P($\beta < 0$)*
Preceding region	NP1 complexity	−0.002	−0.021	0.016	.596
	NP2 complexity	−0.009	−0.026	0.007	.871
	NP1 × NP2 complexity	−0.003	−0.018	0.012	.653
RC verb	NP1 complexity	0.002	−0.013	0.017	.369
	NP2 complexity	−0.019	−0.035	−0.003	.989
	NP1 × NP2 complexity	0.013	−0.004	0.030	.070

1.1 EXPERIMENT 1 MATERIALS

Only the version with complex NP1 and NP2 is shown here:

1. The federal prison warden punished the condemned political prisoner who the guard at the old facility supplied with illegal goods like cigarettes.

2. The important, prospective client harassed the attractive personal secretary who the co-worker at the giant firm accused of drinking on the job.

3. The regular, valued customer trusted the sleazy car salesman who the mechanic at the auto shop helped to hide the engine problems.

4. The experienced stage choreographer embraced the graceful ballet dancer who an admirer in the front row begged for an autograph for his collection.

5. The Marine Corps sergeant dismissed the wounded American soldier who the commander at the army base ordered to search every car that approached the checkpoint.

6. The noisy, obnoxious drunk heckled the hilarious stand-up comedian who the bartender with a strong accent defended from the back of the room.

7. The accident insurance investigator questioned the emotional crash survivor who the pilot with a broken arm pulled from the wreckage of the plane.

8. The brutal military policeman arrested the peaceful Buddhist monk who a councillor in the capital city saved from being imprisoned and tortured.

9. The conservative US congressman interrogated the victorious four-star general who a lawyer for the White House advised to not comment on the prisoners.

10. The young, rebellious teenager hated the struggling rock musician who a scout for a record company discovered several years ago in Paris.

11. The alleged bombing accomplice misled the undercover federal agent who a witness in the terrorism trial notified of suspicious activity outside the Parliament building.

12. The celebrity hair stylist offended the renowned fashion designer who a journalist at the spring exhibition asked about the likely trends for next year.

13. The senior electrical engineer disliked the nerdy computer programmer who a hacker on the company network ridiculed for having pathetic security safeguards.

14. The critical newspaper reviewer applauded the famous young actor who the director of the art film ignored during the opening night festivities.

15. The senior foreign diplomat contacted the ruthless military dictator who the activist from the United Kingdom encouraged to preserve natural habitats and resources.

16. The hard-nosed newspaper reporter interviewed the wealthy teen celebrity who the photographer for the tabloid magazine embarrassed last week at a charity dinner.

17. The capital murder defendant feared the corrupt homicide cop who the judge in the court case silenced after a disturbing courtroom outburst.

18. The hard-working factory employee obeyed the professional shift supervisor who an inspector from the county government cautioned about the poor work conditions.

19. The cable news analyst ridiculed the Labour mayoral candidate who the leader of the political party supported despite the anxiety of other members.

20. The chief CIA interrogator questioned the heartless former mercenary who the commander of the armed rebellion shot in the foot without any explanation.

21. The wanted, dangerous fugitive robbed the rich American tourist who the guide for the London tour warned about straying too far from the group.

22. The wealthy German industrialist threatened the poor Russian peasant who the investor from a foreign land protected without the least bit of hesitation.

23. The powerful business executive infuriated the liberal socialist politician who a lobbyist the oil company bribed to vote for the upcoming bill.

24. The influential legal advisor lectured the handsome blonde prince who the official learning to speak English thanked at the end of the ceremony.

25. The elderly passing pedestrian dodged the angry taxi driver who a bystander near the bus stop identified later on at the police station.

26. The happy, suburban housewife offended the helpful new assistant who the manager on a lunch break called into his office after the incident.

27. The trained hospice nurse consoled the dying elderly patient who the doctor on a double shift forgot due to a lack of sleep.

28. The private office secretary aggravated the young female intern who a partner at the law firm hired less than three weeks ago.

1.2 EXPERIMENT 2 MATERIALS

1. The warden punished the prisoner who the guard at the federal prison supplied with illegal contraband like cigarettes.

2. The client harrassed the secretary who the coworker at the giant firm noticed drinking on the job occasionally.

3. The customer doubted the salesman who the mechanic at the auto shop believed to be a deplorable crook.

4. The housewife visited the priest who the bishop with an intimidating presence commended for a life of dedication to the church.

5. The choreographer embraced the dancer who the admirer in the front row begged for an autograph for his daughter.

6. The sergeant relieved the soldier who the commander of the army troops ordered to search every car that approached the checkpoint.

7. The drunkard heckled the comedian who the bartender on his lunch break defended from the back of the room.

8. The investigator questioned the survivor who the pilot with a broken arm pulled from the burning fuselage.

9. The policeman arrested the monk who the councilor in the capital city saved from being imprisoned and tortured.

10. The congressman interrogated the general who the lawyer for the Bush administration advised to not comment on the detainees.

11. The teenager hated the musician who the girl listening to the song adored since she saw the late night talk show performance.

12. The accomplice misled the agent who the witness in the impeachment trial notified about the presence of two suspicious characters.

13. The stylist offended the designer who the interviewer at the spring exhibition asked about the likely trends for next year.

14. The engineer debated the programmer who the hacker on the company network ridiculed for having pathetic security safeguards.

15. The reviewer criticized the actor who the director of the art film ignored during the opening night festivities.

16. The diplomat contacted the dictator who the activist from the United Kingdom encouraged to preserve natural habitats and resources.

17. The reporter interviewed the celebrity who the photographer for the tabloid magazine embarrassed last week at a charity dinner.

18. The defendant accused the cop who the judge in the murder case silenced after a disturbing courtroom outburst.

19. The guard aided the criminal who the agent from the U.S. marshals apprehended following a long and tiring chase.

20. The employee obeyed the supervisor who the inspector of the safety measures cautioned about the poor work conditions.

21. The captain evaluated the fireman who the veteran with 30 years' experience trained over the course of six months.

22. The investigator summoned the athlete who the coach of the football team invited to try out for a spot on the team.

23. The pundit ridiculed the candidate who the leader of the political party supported despite the misgivings of other members.

24. The interrogator questioned the mercenary who the commander of the armed rebellion abandoned without any explanation or warning.

25. The fugitive robbed the tourist who the guide for the huge group warned about straying too far from the group.

26. The industrialist threatened the peasant who the investor from a foreign land protected without the least bit of hesitation.

27. The executive infuriated the politician who the lobbyist for some oil companies bribed to vote for the upcoming bill.

28. The advisor lectured the prince who the dignitary learning to speak English thanked at the end of the ceremony.

29. The pedestrian dodged the driver who the bystander near the bus stop identified later on at the police station.

30. The customer offended the assistant who the manager of the department store called into his office after the incident.

31. The nurse consoled the patient who the doctor on a double shift forgot due to a lack of sleep.

32. The secretary aggravated the intern who the partner at the law firm hired less than three weeks ago.
